# Owls and Larks in Mice

**DOI:** 10.3389/fneur.2015.00101

**Published:** 2015-05-15

**Authors:** Martina Pfeffer, Helmut Wicht, Charlotte von Gall, Horst-Werner Korf

**Affiliations:** ^1^Dr. Senckenbergische Anatomie II, Fachbereich Medizin der Goethe-Universität, Frankfurt am Main, Germany; ^2^Dr. Senckenbergisches Chronomedizinisches Institut, Fachbereich Medizin der Goethe-Universität, Frankfurt am Main, Germany; ^3^Institut für Anatomie II, Fachbereich Medizin, Heinrich Heine Universität, Düsseldorf, Germany

**Keywords:** circadian rhythms, chronotype, chronobiology, neurodegeneration, stability, activity rhythms

## Abstract

Humans come in different chronotypes and, particularly, the late chronotype (the so-called owl) has been shown to be associated with several health risks. A number of studies show that laboratory mice also display various chronotypes. In mice as well as in humans, the chronotype shows correlations with the period length and rhythm stability. In addition, some mouse models for human diseases show alterations in their chronotypic behavior, which are comparable to those humans. Thus, analysis of the behavior of mice is a powerful tool to unravel the molecular and genetic background of the chronotype and the prevalence of risks and diseases that are associated with it. In this review, we summarize the correlation of chronotype with free-running period length and rhythm stability in inbred mouse strains, in mice with a compromised molecular clockwork, and in a mouse model for neurodegeneration.

For all organisms, the ability to respond and adapt to changes in the environment is important to ensure survival (1, 2). Circadian clocks have evolved to fine tune biological functions to specific times within the day or night (3, 4). A disruption of the time keeping system can lead to physiological and psychological problems that can be experienced during transmeridian air travel (jet lag) and include fatigue, often also gastrointestinal and metabolic problems. The psychological symptoms are poor concentration, decreased attention, and problems in memory formations (5). Also, shift work has severe effects on the human time keeping system, and is associated with reduced cognitive performance and an increased risk of cardiovascular diseases, cancer, and sleep disorders (5–8). In mice, chronic jet lag increases mortality levels and accelerates tumor progression (9, 10).

Under natural conditions, phase and period length of the molecular clockwork are entrained to the environmental day/night cycle; but within a given species, there is a substantial variability in the temporal alignment. In order to describe this phenomenon, the term “chronotype” was coined by Ehret (11), and he provided a very succinct definition of the term: “the temporal phenotype of an organism.” Notably, this definition refers to *overt* characters and not to a propensity or inclination. It is somewhat ambiguous with respect to the word “temporal,” as it does not become clear whether it refers to internal (endogenous/circadian, CT) or external (sidereal/*zeitgeber-*, ZT) time. But, Ehret (11) then clarifies this ambiguity by visualizing the “chronotype of the rat.” He plots the acrophases of various behavioral, metabolic, and endocrine activities over diurnal (*zeitgeber*-) time. He implicitly supplies an operational definition of how the chronotype may be measured – by comparing the timing of these acrophases among each other and in relation to the entraining *zeitgebers*, thus permitting to distinguish “early” and “late” chronotypes.

## Chronotypes in Humans

The Horne–Östberg questionnaire (12) chronotypes humans based on a scoring system (“morningness–eveningness”). It *does* take into account (psychic) factors, propensities, and inclinations that may *not* necessarily manifest themselves in the daily behavior. Roenneberg and colleagues (13, 14), on the other hand, relied on an overt character (the “mid-sleep on free days”) to chronotype large populations of humans.

In humans, the chronotypes differ considerably. If the “mid-sleep on free days” is used to quantify the chronotype (13, 14), extreme “larks” and “owls” differ by more than 8 h. Beyond sleep/wake timing, different chronotypes show distinct and different temporal patterns in cognitive performance, gene expression, and endocrine functions (15–17).

The late chronotypes, in particular, are subject to repeated temporal shifts, due to the fact that they are forced into the behavioral patterns of a lark-society (15, 18). The term “social jetlag” has been introduced to denote this shifting entrainment that often results in chronic sleep loss (19, 20). Furthermore, a late chronotype is associated with a multitude of health risks, such as metabolic dysfunctions, obesity, depression, sleep disturbances, and nicotine abuse (15, 16, 21). Social jetlag is also associated with endocrine and cardiovascular risks (22), and negatively correlates with academic performance in undergraduates (23).

Mice might be useful to elucidate the genetic, cellular, and molecular mechanisms that underlie the association between chronotype, time-shifts, health, and disease – if they could be chronotyped. In fact, they can.

## Chronotypes in Mice

Most mouse strains used in laboratory research are nocturnal. Under entrained conditions, i.e., under a 12 h light/12 h dark (LD) regime, and food and water *ad libitum*, “lights off” marks the onset of the main activity period (24–28). However, there are also mice that become active already in the light phase, several hours before “lights off” (29).

These results suggest that different mouse strains have different chronotypes. The “mid sleep on free days,” used to chronotype humans, cannot be determined in mice, since their sleep-wake cycles differ radically from those of humans. Instead of having a single continuous sleep phase in a 24 h-interval, mice have numerous short periods of sleep throughout the 24 h cycle (30–32). Furthermore, the activity profiles of mice are not smooth, but show numerous “spikes” and “depressions”; thus, attempts to chronotype mice by calculating the acrophases of their locomotor activity failed to distinguish between the different strains.

Alternatively, chronotypes in mice may be quantified by subtracting the time [ZT] of the occurrence of the entraining cue (i.e., “lights off” in a light entrainment regime) from the time of the onset, the main activity phase. This time span is slightly positive in most nocturnal mouse strains, which become active shortly after “lights off,” and negative in the few ones (29) that become active before the onset of darkness. This method does, however, not permit to reliably distinguish chronotypes between the most widely used strains of mice, and it also will not quantify the obvious differences in the temporal dynamics of the activity profiles that occur after the onset of activity. Some mice (C3H/HeN (C3H)) have a single activity peak early in the night, while others (C57Bl/6J (C57Bl/6)), extend their activity well into the early morning.

**Figure 1:**
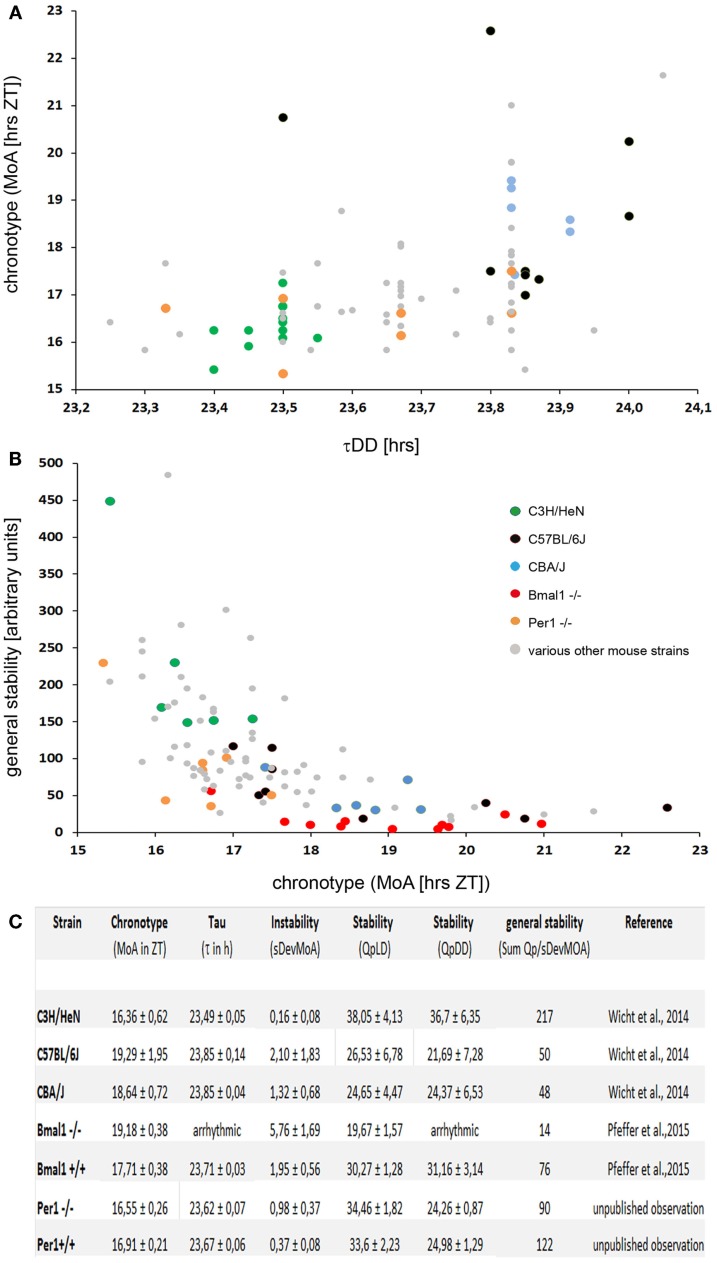
**(A)** A plot of the τDD vs. chronotype (MoA) in 89 individual mice of various strains. Some of the strains mentioned in the text are highlighted by colored dots, see insert in **(B)** for the code. The MoA was determined as described in Ref. (28). There is a moderate correlation of rho = 0.47 (Spearman’s rank correlation) between the two parameters. Note that the differences in the τDD are less than an hour, while the chronotypes differ by more than 6 h. **(B)** A plot of the chronotype (MoA) vs. the general stability of the locomotor rhythms in 103 individual mice of various strains. The general stability is scaled in arbitary units; high numbers indicate stable rhythms. The numbers were calculated as described in Ref. (28), in short; they represent a combined measure of stability using Refinetti’s (39) Qp-values and the accuracy with which the individual mice reproduce their chronotype in a day-to-day comparison. Note that the Bmal1^−/−^ mice (red dots) which do not show up in **(A)**, as they have no τDD, do have a (late) chronotype. Yet, they are not the least stable mice; some individuals of the C57BL/6 strain have even less stable locomotor rhythms. There is a good correlation of rho = -0.75 (Spearman’s rank correlation) between the two parameters. **(C)** Table with the chronotype (MoA), instability (sDevMoA), stability (QpLD and QpDD), and general stability of several mouse strains measured so far. The “general stability” [in **(B)**] is calculated by dividing the sum of the Qps (QpLD and QpDD) by the SDev of the MoA.

In search for a parameter that defines the chronotype of mice, it was found that cumulative plots of the activity profiles allow reproducible measurements of the chronotype by determining the “median of activity” (MoA) defined by spontaneous locomotor activity of the mice kept under a standard 12/12 LD regime (28). The MoA is the time-point on the timescale at which the mouse has completed 50% of its daily “activity work” under entrained conditions. “Early” mice will have accomplished that “task” at an earlier time (ZT) than “late” ones. Indeed, the inbred mouse strains that are routinely used in circadian research (33–38), namely the C3H, C57Bl/6 and CBA/J mice, do have significantly different chronotypes, even though they do not differ with regard to the onset of the activity phase: the C57Bl/6 are the “owls” among them, the C3H are the “larks,” and the CBA/J are of intermediate type (Figure 1).

Notably, the MoA – in its current definition (28) – can only be applied to nocturnal strains of mice (see above). The starting point of the cumulative plots that are used to determine the 50% values is currently fixed at ZT 12. If this “fixed starting point” procedure was used to chronotype the “early runner” mice (29), the results would obviously be nonsensical, as the calculus would result in a late chronotype. However, this weakness can be overcome by combining the MoA with Wisor et al.’s (29) method of determining the onset of the main activity phase in hours ZT and starting the cumulative procedure at that point in time. The MoA – like any other chronotyping method – can only be applied to animals that do entrain to external stimuli, arrhythmicity, and chronotypic behavior are mutually exclusive.

## Free-Running Period Length, Molecular Clockwork, and Chronotype

Rhythmic behavior persists even in constant darkness (DD), although with a period slightly shorter or longer than that of the 24-h-day, demonstrating that the free-running oscillation is the output of an endogenous temporal program. The length of the period under DD is denoted by the Greek letter τ (tau), followed by a “DD.” The τDD is thought to be mainly determined by the endogenous clock and clock genes, the key components of the molecular clockwork (40).

Indeed, mice carrying mutations or targeted deletions of clock genes, such as Clock, Cry, or Per (41–47), either show a dramatic change in the length of their τDD or become arrhythmic under constant conditions [Bmal1; (48)]. In humans, a missense mutation in the clock gene hPER2 is associated with a variant in human sleep behavior, the familial advanced sleep phase syndrome (FASPS), which is characterized by a significantly shorter period length of circadian rhythms (49). However, the clock-genes are certainly *not* the sole genes that determine the τDD. If the Per-1 gene is knocked out in C57BL/6 mice, their τDD is 1 h shorter than that of their wild-type littermates (47). If the very same gene – Per-1 – is deleted in C3H mice (50), the τDD is not affected (Figure 1).

It was generally assumed that there is a strict correlation between the τDD and the chronotype; however, recent studies have shown that this is not always the case. In finches and humans, a correlation between τDD and chronotype has been demonstrated (49, 51–53). Also, in laboratory mice, the chronotype correlates with the τDD; C57Bl/6 mice have a longer τDD and a later chronotype as compared to C3H mice, but the correlation between the τDD and the chronotype is quite moderate [(28); Figure 1]. Notably, this correlation between the τDD and the chronotype is absent in some cases: In Cry1 KO as well as in Cry2 KO mice, the chronotypic differences in LD (measured by the onset of activity relative to lights-off) are minimal compared to their background-matched wild-types, despite their large differences in free-running periods (54). In accordance with these observations, Wisor et al. (29) report the absence of a correlation between the τDD and the onset of the activity period in their “early runner” mice.

The BMAL1 KO (Bmal1^−/−^), a mouse with a defective molecular clockwork, is arrhythmic in constant darkness, and therefore has no τDD (48). But under entrained conditions, these animals do display a rhythmic behavior and a chronotype. It is later than that of their wild-type littermates (C57Bl/6 background), and they display a less stable rhythm (55). It seems that the chronotype and the τDD are not correlated in a simple and predictable manner. In fact, a τDD is not even necessary in order to have a chronotype.

It should also be noted that – in terms of absolute duration – the τDDs of the different mouse strains differ by about 20–40 min, while the differences between the chronotypes amount to several hours [see Figure 1; (24–29, 55)]. Furthermore, the τDD cannot be causal for the establishment of the chronotype – simply because the chronotype manifests itself only under entrained conditions, while the τDD requires free-running (non-entrained) conditions in order to be realized. Under conditions of normal nycthemeral entrainment, however, the period length of the behavioral rhythms will be 24.00 h. These data suggest that the chronotype is influenced by genetic factors, and many of them probably reside outside the classical clock genes as shown for several mouse strains (24–27, 29).

## Chronotype and Rhythm Stability

Different mouse strains also differ with respect to the inter- and intra-individual variances and the stability of the behavioral rhythms. The timely precision with which mice (and other beings) reproduce their behavioral rhythms under different conditions (be they entrained or free-running) can be quantified using a combination of different methods [(28, 39); and Figure 1]. The standard deviation of the MoA (SDevMoA) is a measure of the rhythm instability, which quantifies the more or less pronounced daily variation of the activity pattern. In contrast, the Qp-analysis quantifies the rhythm stability in LD and in DD by estimating the stability and robustness of an animal’s τ over several periods as compared to an ideal, sinusoidal wave with the same wavelength (39). Both measures correlate inversely and were used to calculate the “general stability” by simply dividing the summed Qps (LD and DD) for each mouse by the SDev of its MoA [(28); Figure 1].

Interestingly, a quite robust correlation exists between rhythm stability in locomotor activity and the chronotype (Figure 1). This could be observed in all mouse strains examined so far – mice with an early chronotype reproduce their rhythms (both free-running and entrained) more precisely than those with a late one (28, 55). The factors constituting the causal link for the correlation between chronotype and rhythm stability are currently unknown.

A similar correlation between chronotypes and rhythm stability has been found in humans (13, 20). But in humans, the rhythmic instability of the late chronotypes (“owls”) is attributed to the repeated shifts in the (social) entrainment that they suffer – the phase of entrainment on their “work days” differs (more than in “larks”) from their phase on “free days,” while the stability differences in mice were observed under conditions of constant, non-shifting entrainment.

Researches into the stability (or lability) of circadian and diurnal rhythms have a long tradition [see, for example, Ref. (56)]. In spite of the presence of a plethora of data on various species under varying conditions, the biological role of rhythm stability vs. lability is still unknown.

## Chronotype, Entrainment, and Disease

The photoperiod has been shown to influence the chronotype in humans – short days (in winter) will shift the average chronotype of a population to a later while long days (in summer) will shift it to an earlier one (57, 58). A similar effect can be observed in mice. Under short days and long nights, their night activity profiles will become “expanded” – i.e., “later,” if the “MoA” were applied (39). Inversely, short nights will compress the activity profiles resulting in an earlier chronotype. Notably, the different photoperiods do *not* affect the onset of activity in relation to the onset of darkness (39). However, not only the duration of light exposure affects the chronotype but also the capacity of an individual to entrain to a certain light stimulus.

**Figure 2:**
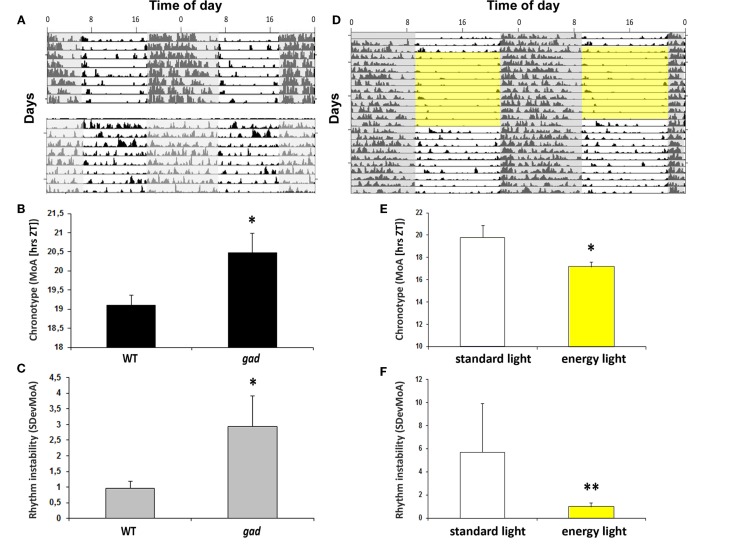
**(A)** Actograms of locomotor activity from a representative gad mouse (lower actogram) and a WT littermate (upper actogram). **(B)** Bar plot of the chronotype (MoA) as a measurement for chronotype. Data are expressed as the mean ± SEM (*n* = 8); *P < 0.05. **(C)** Bar plot of sDevMoA as a measurement of rhythm instability. Data are expressed as the mean ± SEM (*n* = 8); *P < 0.05 **(D)** Actogram of locomotor activity in 12 h “energy light”/12 h darkness (BL, 7.500 lux, Energy light, Philips Healthcare, Germany; yellow square) and in a standard photoperiod from a representative *gad* mouse. **(E)** Bar plot of the chronotype (MoA) of gad mice exposed to “*energy light*” (indicated by the yellow box) or “regular” light during the light phase. Data are expressed as the mean ± SEM (*n* = 4), *P < 0.05 **(F)** Bar plot of rhythm instability (sDevMoA) of *gad* mouse exposed to “energy” or “regular” light during the light phase. Data are expressed as the mean ± SEM (*n* = 4). Even the rhythm instability is not significant between *gad* and WT mice, the difference between the variances are significant. **P < 0.001.

Aged mPer1/mCry2 mutant mice lose their rhythmic wheel-running behavior under LD conditions. This loss is caused by an impaired light signal transduction (59). This suggests that an impairment of circadian light perception and processing can be associated with the rhythm instability and the chronotype. Indeed, many neurodegenerative diseases such as Parkinson’s or Alzheimer’s disease are associated with sleep disturbances (60) presumably as a consequence of disturbances of proteostasis, resulting in a compromised circadian clock (61). However, in the gracile axonal dystrophy (*gad*) mice with a spontaneous mutation in the Park5 gene which encodes for ubiquitin carboxy-terminal hydrolase L1 (UCHL1), the endogenous rhythm generator in the SCN stays intact. The τDD is thus not affected (62), but the chronotype is – in comparison to the wild-type littermates – shifted to a later point in time. Along with the change in chronotype (Figure 2B), the mice show an increasingly unstable rhythm (Figures 2A,C). The *gad* mice suffer from a loss of the circadian photopigment (melanopsin) in the retina (62), which may be responsible for changes in the chronotype and the development of rhythm instability. Notably, the chronotype became earlier, and rhythm instability was less variable at higher luminance levels (Figures 2E,F). This is consistent with reduced daytime activity at higher irradiance levels (Figure 2D; 62). Thus, impaired circadian light perception as a consequence of neurodegeneration resulting in circadian disruption was rescued by light “therapy” to some extent.

In addition, age also affects the chronotype. In humans, the chronotype becomes progressively later during adolescence and then gradually shifts to an earlier chronotype again (63). Also in mice, there is evidence for a pubertal shift in the timing of daily rhythms (64). The circadian organization changes with further aging in mice including fragmentation of the activity rhythm and a decreased precision in onset of daily activity (65). It is most likely that these changes seen in the activity rhythm are accompanied by a change in the chronotype, even though this has not been analyzed yet numerically. This indicates that that the chronotype is not static and may be influenced by external and internal factors.

## Conclusion

With respect to the current researches on that topic, Ehret’s (11) definition of the chronotype should be expanded to “the temporal phenotype of an organism in relation to entraining cues (*zeitgebers*)”, in particular, since it has become clear that behavioral characters that are observed under (artificial) constant conditions (i.e., in the absence of external *zeitgebers*) are of restricted predictive value with respect to entrained behavior, and also that the chronotype is malleable and can be influenced by external or internal changes. Therefore, the chronotype is the overt behavioral manifestation of the interplay of external and internal factors that makes us more or less susceptible to problems and diseases that arise from the “false timing.” Laboratory mice offer the opportunity to analyze this interplay under controlled external (entrainment) and internal (genetic) conditions. Furthermore, they provide an excellent model system to develop new therapeutical strategies to alleviate chronodisruption.

## Conflict of Interest Statement

The authors declare that the research was conducted in the absence of any commercial or financial relationships that could be construed as a potential conflict of interest.

## References

[1] Darwin C On The Origin of Species by Means of Natural Selection. 1st Edn, Vol. 108, Chap. 4. London: John Murray (1859).

[2] Sharma VK. Adaptive significance of circadian clocks. Chronobiol Int (2003) 20:901–19. 10.1081/CBI-120026099 14680135

[3] Hastings MH Brancaccio M Maywood ES. Circadian pacemaking in cells and circuits of the suprachiasmatic nucleus. J Neuroendocrinol (2014) 26:2–10. 10.1111/jne.12125 24329967 PMC4065364

[4] Korf HW von Gall C Circadian physiology. In: Pfaff DW, editor. Textbook of Neuroscience in the 21st Century: Basic and Clinical. New York: Springer (2012). p. 1813–45.

[5] Cho K Chronic “jetlag” produces temporal lobe atrophy and spatialcognitive deficits. Nat Neurosci (2001) 4:567–8 10.1038/88384 11369936

[6] Arendt J. Shift work: coping with the biological clock. Occup Med (2010) 60:10–20. 10.1093/occmed/kqp162 20051441

[7] Cho K Ennaceur A Cole JC Suh CK. Chronic jet lag produces cognitive deficits. J Neurosci (2000) 20:RC66 10704520 10.1523/JNEUROSCI.20-06-j0005.2000 PMC6772481

[8] Touitou Y. Adolescent sleep misalignment: a chronic jet lag and a matter of public health. J Physiol (2013) 107:323–6. 10.1016/j.jphysparis.2013.03.008 23542542

[9] Davidson AJ Sellix MT Daniel J Yamazaki S Menaker M Block GD Chronic jet-lag increases mortality in aged mice. Curr Biol (2006) 16:R914–6 10.1016/j.cub.2006.09.058 17084685 PMC1635966

[10] Filipski E Delaunay F King VM Wu MW Claustrat B Grechez-Cassiau A Effects of chronic jet lag on tumor progression in mice. Cancer Res (2004) 64:7879–85. 10.1158/0008-5472.CAN-04-0674 15520194

[11] Ehret CF The sense of time: evidence for its molecular basis in the eukaryotic gene-action system. Adv Biol Med Phys (1974) 15:47–77 10.1016/B978-0-12-005215-8.50009-7 4600894

[12] Horne JA Östberg O. A self-assessment questionnaire to determine morningness-eveningness in human circadian rhythms. Int J Chronobiol (1975) 4:97–110. 1027738

[13] Roenneberg T Wirz-Justice A Merrow M. Life between clocks: daily temporal patterns of human chronotypes. J Biol Rhythms (2003) 18:80–90. 10.1177/0748730402239679 12568247

[14] Zavada A Gordijn MC Beersma DG Daan S Roenneberg T. Comparison of the Munich chronotype questionnaire with the Horne-Ostberg’s morningness-eveningness score. Chronobiol Int (2005) 22:267–78. 10.1081/CBI-200053536 16021843

[15] Roenneberg T Allebrandt KV Merrow M Vetter C Social jetlag and obesity. Curr Biol (2012) 22:939–43 10.1016/j.cub.2012.03.038 22578422

[16] Foster RG Peirson SN Wulff K Winnebeck E Vetter C Roenneberg T. Sleep and circadian rhythm disruption in social jetlag and mental illness. Prog Mol Biol Transl Sci (2013) 119:325–46. 10.1016/B978-0-12-396971-2.00011-7 23899602

[17] Juda M Vetter C Roenneberg T. Chronotype modulates sleep duration, sleep quality, and social jet lag in shift-workers. J Biol Rhythms (2013) 28:141–51. 10.1177/0748730412475042 23606613

[18] Merrow M Spoelstra K Roenneberg T. The circadian cycle: daily rhythms from behaviour to genes. EMBO Rep (2005) 10:930–5. 10.1038/sj.embor.7400541 16222241 PMC1369194

[19] Roenneberg T Kuehnle T Juda M Kantermann T Allebrandt K Gordijn M Epidemiology of the human circadian clock. Sleep Med Rev (2007) 11:429–38. 10.1016/j.smrv.2007.07.005 17936039

[20] Wittmann M Dinich J Merrow M Roenneberg T. Social jetlag: misalignment of biological and social time. Chronobiol Int (2006) 23:497–509. 10.1080/07420520500545979 16687322

[21] Golombek DA Casiraghi LP Agostino PV Paladino N Duhart JM Plano SA The times they’re a-changing: effects of circadian desynchronization on physiology and disease. J Physiol (2013) 107:310–22. 10.1016/j.jphysparis.2013.03.007 23545147

[22] Rutters F Lemmens SG Adam TC Bremmer MA Elders PJ Nijpels G Is social jetlag associated with an adverse endocrine, behavioral, and cardiovascular risk profile? J Biol Rhythms (2014) 29:377–83. 10.1177/0748730414550199 25252710

[23] Haraszti RA Ella K Gyongyosi N Roenneberg T Kaldi K. Social jetlag negatively correlates with academic performance in undergraduates. Chronobiol Int (2014) 31:603–12. 10.3109/07420528.2013.879164 24491157

[24] Hofstetter JR Mayeda AR Possidente B Nurnberger JI Jr. Quantitative trait loci (QTL) for circadian rhythms of locomotor activity in mice. Behav Genet (1995) 25:545–56. 10.1007/BF02327578 8540893

[25] Hofstetter JR Trofatter JA Kernek KL Nurnberger JI Mayeda AR. New quantitative trait loci for the genetic variance in circadian period of locomotor activity between inbred strains of mice. J Biol Rhythms (2003) 18:450–62. 10.1177/0748730403259468 14667146

[26] Suzuki T Ishikawa A Nishimura M Yoshimura T Namikawa T Ebihara S. Mapping quantitative trait loci for circadian behavioral rhythms in SMXA recombinant inbred strains. Behav Genet (2000) 30:447–53. 10.1023/A:1010298701251 11523704

[27] Shimomura K Low-Zeddies SS King DP Steeves TD Whiteley A Kushla J Genome-wide epistatic interaction analysis reveals complex genetic determinants of circadian behavior in mice. Genome Res (2001) 11:959–80. 10.1101/gr.171601 11381025

[28] Wicht H Korf HW Ackermann H Ekhart D Fischer C Pfeffer M Chronotypes and rhythm stability in mice. Chronobiol Int (2014) 1:27–36 10.3109/07420528.2013.820739 24079808

[29] Wisor JP Striz M DeVoss J Murphy GM Jr Edgar DM O’Hara BF. A novel quantitative trait locus on mouse chromosome 18, “era1,” modifies the entrainment of circadian rhythms. Sleep (2007) 30:1255–63. 17969459 10.1093/sleep/30.10.1255 PMC2266285

[30] Mitler MM Lund R Sokolove PG Pittendrigh CS Dement WC. Sleep and activity rhythms in mice: a description of circadian patterns and unexpected disruptions in sleep. Brain Res (1977) 131:129–45. 10.1016/0006-8993(77)90033-6 195675

[31] Daszuta A Gambarelli F Ternaux JP. Sleep variations in C57BL and BALBc mice from 3 weeks to 14 weeks of age. Brain Res (1983) 283:87–96. 10.1016/0165-3806(83)90084-6 6831259

[32] Veasey SC Valladares O Fenik P Kapfhamer D Sanford L Benington J An automated system for recording and analysis of sleep in mice. Sleep-New York (2000) 23:1025–42. 11145318

[33] von Gall C Stehle JH Weaver DR. Mammalian melatonin receptors: molecular biology and signal transduction. Cell Tissue Res (2002) 309:151–62. 10.1007/s00441-002-0581-4 12111545

[34] Gau D Lemberger T von Gall C Kretz O Le Minh N Gass P Phosphorylation of CREB Ser142 regulates light-induced phase shifts of the circadian clock. Neuron (2002) 11:245–53. 10.1016/S0896-6273(02)00656-6 11970866

[35] Korf HW von Gall C. Mice, melatonin and the circadian system. Mol Cell Endocrinol (2006) 252:57–68. 10.1016/j.mce.2006.03.005 16644097

[36] Ono H Hoshino Y Yasuo S Watanabe M Nakane Y Murai A Involvement of thyrotropin in photoperiodic signal transduction in mice. Proc Natl Acad Sci U S A (2008) 105:18238–42. 10.1073/pnas.0808952105 19015516 PMC2587639

[37] Yasuo S Yoshimura T Ebihara S Korf HW. Melatonin transmits photoperiodic signals through the MT1 melatonin receptor. J Neurosci (2009) 29:2885–9. 10.1523/JNEUROSCI.0145-09.2009 19261884 PMC6666200

[38] Pfeffer M Rauch A Korf H-W von Gall C. The endogenous melatonin signal facilitates reentrainment of the circadian system to light-induced phase advances by acting upon MT2 receptors. Chronobiol Int (2012) 29:415–29. 10.3109/07420528.2012.667859 22489607

[39] Refinetti R. Non-stationary time series and the robustness of circadian rhythms. J Theor Biol (2004) 227:571–81. 10.1016/j.jtbi.2003.11.032 15038991

[40] Reppert SM Weaver DR. Coordination of circadian timing in mammals. Nature (2002) 418:935–41. 10.1038/nature00965 12198538

[41] Bae K Jin X Maywood ES Hastings MH Reppert SM Weaver DR Differential functions of mPer1, mPer2, and mPer3 in the SCN circadian clock. Neuron (2001) 30:525–36 10.1016/S0896-6273(01)00302-6 11395012

[42] DeBruyne JP Weaver DR Reppert SM. CLOCK and NPAS2 have overlapping roles in the suprachiasmatic circadian clock. Nat Neurosci (2007) 10:543–5. 10.1038/nn1884 17417633 PMC2782643

[43] van der Horst GT Muijtjens M Kobayashi K Takano R Kanno S Takao M Mammalian Cry1 and Cry2 are essential for maintenance of circadian rhythms. Nature (1999) 398:627–30. 10.1038/19323 10217146

[44] Sun ZS Albrecht U Zhuchenko O Bailey J Eichele G Lee CC. RIGUI, a putative mammalian ortholog of the *Drosophila* period gene. Cell (1997) 90:1003–11. 10.1016/S0092-8674(00)80366-9 9323128

[45] Vitaterna MH King DP Chang AM Kornhauser JM Lowrey PL McDonald JD Mutagenesis and mapping of a mouse gene, clock, essential for circadian behavior. Science (1994) 264:719–25. 10.1126/science.8171325 8171325 PMC3839659

[46] Wilsbacher LD Sangoram AM Antoch MP Takahashi JS The mouse clock locus: sequence and comparative analysis of 204 Kb from mouse chromosome 5. Genome Res (2000) 10:1928–40 10.1101/gr.155400 11116088 PMC313079

[47] Zheng B Larkin DW Albrecht U Sun ZS Sage M Eichele G Lee CC Bradley A The mPer2 gene encodes a functional component of the mammalian circadian clock. Nature (1999) 400:169–73. 10.1038/22118 10408444

[48] Bunger MK Wilsbacher LD Moran SM Clendenin C Radcliffe LA Hogenesch JB Mop3 is an essential component of the master circadian pacemaker in mammals. Cell (2000) 103:1009–17. 10.1016/S0092-8674(00)00205-1 11163178 PMC3779439

[49] Toh KL Jones CR He Y Eide EJ Hinz WA Virshup DM An hPer2 phosphorylation site mutation in familial advanced sleep phase syndrome. Science (2001) 291:1040–3. 10.1126/science.1057499 11232563

[50] Christ E Pfeffer M Korf HW von Gall C. Pineal melatonin synthesis is altered in Period1 deficient mice. Neuroscience (2010) 171:398–406. 10.1016/j.neuroscience.2010.09.009 20849936

[51] Aschoff J Wever R Über Phasenbeziehungen zwischen biologischer Tagesperiodik und Zeitgeberperiodik. Z Vgl Physiol (1962) 46:115–28 10.1007/BF00341546

[52] Duffy JF Rimmer DW Czeisler CA. Association of intrinsic circadian period with morningness-eveningness, usual wake time, and circadian phase. Behav Neurosci (2001) 115:895–9. 10.1037/0735-7044.115.4.895 11508728

[53] Brown SA Kunz D Dumas A Westermark PO Vanselow K Tilmann-Wahnschaffe A Molecular insights into human daily behavior. Proc Natl Acad Sci U S A (2008) 105:1602–7. 10.1073/pnas.0707772105 18227513 PMC2234191

[54] Spoelstra K Albrecht U van der Horst GTJ Brauer V Daan S. Phase responses to light pulses in mice lacking functional per or cry genes. J Biol Rhythms (2004) 6:518–29. 10.1177/0748730404268122 15523113

[55] Pfeffer M Korf HW von Gall C. Chronotype and stability of spontaneous locomotor activity rhythm in BMAL1-deficient mice. Chronobiol Int (2015) 32:81–91. 10.3109/07420528.2014.956218 25216070

[56] Pittendrigh CS Daan S. A functional analysis of circadian pacemakers in nocturnal rodents. J Comp Physiol (1976) 106:223–52. 10.1007/BF0141786010802100

[57] Murray G. Allen NB. Trinder J Seasonality and circadian phase delay: prospective evidence that winter lowering of mood is associated with a shift towards Eveningness. J. Affective Disorders (2003) 76:15–22 10.1016/S0165-0327(02)00059-9 12943929

[58] Allebrandt KV Teder-Laving M Kantermann T Peters A Campbell H Rudan I Chronotype and sleep duration: The influence of season of assessment. Chronobiol. Int, (2014) 31, 731–40 10.3109/07420528.2014.901347 24679223

[59] Oster H Baeriswyl S Van Der Horst GT Albrecht U. Loss of circadian rhythmicity in aging mPer1-/- mCry2-/- mutant mice. Genes Dev (2003) 17:1366–79. 10.1101/gad.256103 12782655 PMC196069

[60] Dauvilliers Y Insomnia in patients with neurodegenerative conditions. Sleep Med (2007) 8:27–34 10.1016/S1389-9457(08)70006-6 18346674

[61] Hastings MH Goedert M. Circadian clocks and neurodegenerative diseases: time to aggregate? Curr Opin Neurobiol (2013) 23:880–7. 10.1016/j.conb.2013.05.004 23797088 PMC3782660

[62] Pfeffer M Plenzig S Gispert S Wada K Korf HW von Gall C. Disturbed sleep/wake rhythms and neuronal cell loss in lateral hypothalamus and retina of mice with a spontaneous deletion in the ubiquitin carboxyl-terminal hydrolase L1 gene. Neurobiol Aging (2012) 33:393–403. 10.1016/j.neurobiolaging.2010.02.019 20363052

[63] Roenneberg T Kuehnle T Pramstaller PP Ricken J Havel M A marker for the end of adolescence. Curr Biol (2004) 14:R1038–9 10.1016/j.cub.2004.11.039 15620633

[64] Weinert D Waterhouse J. Daily activity and temperature rhythms do not change spontaneously with age in laboratory mice. Physiol Behav (1999) 66:605–12. 10.1016/S0031-9384(98)00342-4 10386904

[65] Valentinuzzi VS Scarbrough K Takahashi JS Turek FW. Effects of aging on the circadian rhythm of wheelrunning activity in C57BL/6 mice. Am J Physiol (1997) 273:R1957–64. 9435649 10.1152/ajpregu.1997.273.6.R1957

